# Dr. Clayton E. Spire

**Published:** 1917-07

**Authors:** 


**Dr. Clayton E. Spire,** Cleveland 1897, of Jordan, N. Y., aged 40, was killed by a train striking his automobile, May 17. (Of 45 deaths of physicians listed in the Jour. of the A. M. A., June 9, 3 were due to train-automobile accidents, 1 to street car, 1 to suicide by gun shot and 1 to overdose of morphine).

